# Factors associated with outcomes after reverse total shoulder arthroplasty at midterm follow-up

**DOI:** 10.1016/j.jsea.2026.100074

**Published:** 2026-08-11

**Authors:** Lorenz Fritsch, Brendon C. Mitchell, Michael Nocek, Marilee P. Horan, Grant J. Dornan, Trey C. Rogers, Katelyn L. Kirwood, Shane Corber, Peter J. Millett

**Affiliations:** a The Steadman Philippon Research Institute, Vail, CO, USA; b Department of Sports Orthopaedics, Technical University of Munich, Munich, Germany; c The Steadman Clinic, Vail, CO, USA

**Keywords:** Reverse total shoulder arthroplasty, Patient-reported outcomes, Dominant shoulder, Sex differences, Midterm follow-up, Implant survival

## Abstract

**Background:**

Reverse total shoulder arthroplasty has become the preferred treatment for many patients with irreparable cuff tears, osteoarthritis, and complex proximal humeral fractures. While outcomes are generally favorable, the influence of demographic and surgical factors—such as age, sex, previous surgery, and dominance of the operated arm—on post-operative results remains incompletely understood.

**Methods:**

After institutional review board approval, a retrospective review of prospectively collected data was conducted for all primary reverse total shoulder arthroplasties performed by a single surgeon between December 2011 and December 2022, with a minimum follow-up of 24 months. Typical characteristics, including sex, age at the time of surgery, previous rotator cuff surgery, and subscapularis repair, were recorded. Patient-reported outcome measures included the American Shoulder and Elbow Surgeons (ASES) score, visual analog scale for pain, Single Assessment Numeric Evaluation (SANE), Quick Disabilities of the Arm, Shoulder and Hand (QuickDASH), and 12-Item Short Form Health Survey physical and mental component summaries. These were obtained pre- and post-operatively. Minimal Clinically Important Difference and patient acceptable symptom state for ASES were assessed. Additionally, fatty infiltration was assessed pre-operatively according to the Goutallier classification. Multivariable linear regression analyses were used to identify factors associated with post-operative outcomes.

**Results:**

Final follow-up was obtained for 148 of 185 patients (80%) at a mean of 76.1 ± 32.5 months. The mean age at surgery was 66.0 ± 9.8 years; 61.5% were male. Significant post-operative improvements were observed in ASES, visual analog scale, SANE, QuickDASH, and 12-Item Short Form Health Survey Physical Component Summary scores (all *P* < .05). A total of 90.7% achieved the Minimal Clinically Important Difference for ASES, and 68.9% achieved the patient acceptable symptom state. Male sex and surgery on the dominant arm were associated with superior QuickDASH, and female sex was associated with inferior SANE outcomes (*P* < .05), while age, subscapularis repair, and fatty infiltration showed no significant influence (*P* > .05). The overall failure rate was 4.7%, with dislocation being the most frequent cause (2.7%). The complication rate was 1.3% (2 cases of acromial stress fracture).

**Conclusion:**

Reverse total shoulder arthroplasty provides consistent and durable clinical improvement, with high patient satisfaction and low complication and failure rates. Procedures performed on the dominant arm may yield more favorable outcomes, whereas the female sex has been associated with inferior results in select domains, but without influencing overall failure rates.

Reverse total shoulder arthroplasty (rTSA) has emerged as the primary treatment option for rotator cuff arthropathy, massive irreparable rotator cuff tears, complex proximal humeral fractures, revision of anatomic shoulder arthroplasty, and glenohumeral osteoarthritis with severe bony deformity.^8^^,^^14^^,^^24^ Numerous studies have demonstrated significant improvements in pain relief and function following rTSA,^3^^,^^28^ with low failure and complication rates even at midterm to long-term follow-up.^4^^,^^19^^,^^23^

Several factors, including sex, body mass index, and history of prior shoulder surgery, have been identified as predictors of post-operative outcomes and complication rates.^2^^,^^13^^,^^17^^,^^28^ Younger age and a history of prior rotator cuff surgery are associated with a higher risk of failure and poorer outcomes following rTSA.^9^^,^^22^^,^^25^ Female patients tend to experience less favorable functional outcomes overall, while male patients demonstrate higher rates of revision surgery.^2^^,^^10^^,^^18^^,^^30^

The effect of surgery on the dominant extremity on midterm to long-term results has not been extensively described.^5^^,^^27^ Existing literature on anatomic total shoulder arthroplasty suggests that procedures performed on the dominant side are associated with improved post-operative range of motion but similar functional outcomes.^11^ Nevertheless, the impact of surgery on the dominant shoulder in the context of rTSA remains unclear and is rarely described.^29^

The purpose of this study was to identify factors associated with improved clinical outcomes and implant survival after rTSA at midterm follow-up in a single-surgeon cohort. We hypothesized that rTSA would result in significant improvement in functional outcomes, and that age at implantation would not affect outcomes or failure rates. In contrast, male sex and less fatty degeneration of the teres minor would be associated with better post-operative results.

## Methods

We conducted a retrospective study of consecutively enrolled and prospectively collected data, approved by the institutional review board, WCG Institutional Review Board Protocol #20252021. All primary rTSAs performed by a single surgeon between December 2011 and December 2022 were included. Exclusion criteria were prior arthroplasty and rTSA for failed fracture treatment.

The minimum follow-up period was 24 months.

### Surgical technique

Until 2013, the DeltaXtend reverse shoulder prosthesis system (DePuy Synthes, Raynham, Massachusetts, USA) with a neck-shaft angle of 145° was used. Beginning in 2013, implantation was switched to the Univers Revers Total Shoulder System with the Apex stem (Arthrex, Naples, Florida, USA), featuring a neck-shaft angle of 135°. In all cases treated with the Arthrex system, a standardized glenosphere lateralization of +4 mm was applied. In contrast, no standardized lateralization was used in the DeltaXtend cohort.

The patient was positioned in a slightly inclined beach chair position, and the operative arm was stabilized using an electronic arm holder. A standard deltopectoral approach was utilized. Dissection was carried out on the joint capsule, and the subscapularis tendon was released using a peel-off technique. If the tendon was torn or demonstrated advanced degeneration, a tenotomy was performed. The humerus was then dislocated, and an osteotomy of the humeral head was performed. Preliminary shaft preparation was subsequently carried out.

The synovium was removed, and the glenoid was prepared according to standard technique. An appropriately sized glenosphere was implanted. Final humeral preparation was then performed until a suitable press-fit humeral stem, cup, and inlay components were selected, and sutures for the subsequent subscapularis repair were applied. Trial reduction was carried out, and intraoperative stability was carefully assessed. Vancomycin powder was applied to the intramedullary canal. The final implants were inserted, and the rTSA was reduced.

If the subscapularis tendon was of adequate quality, it was repaired using a modified Mason-Allen technique (Fig. 1, *A-F*). Layered wound closure was performed in standard fashion.

**Figure 1:**
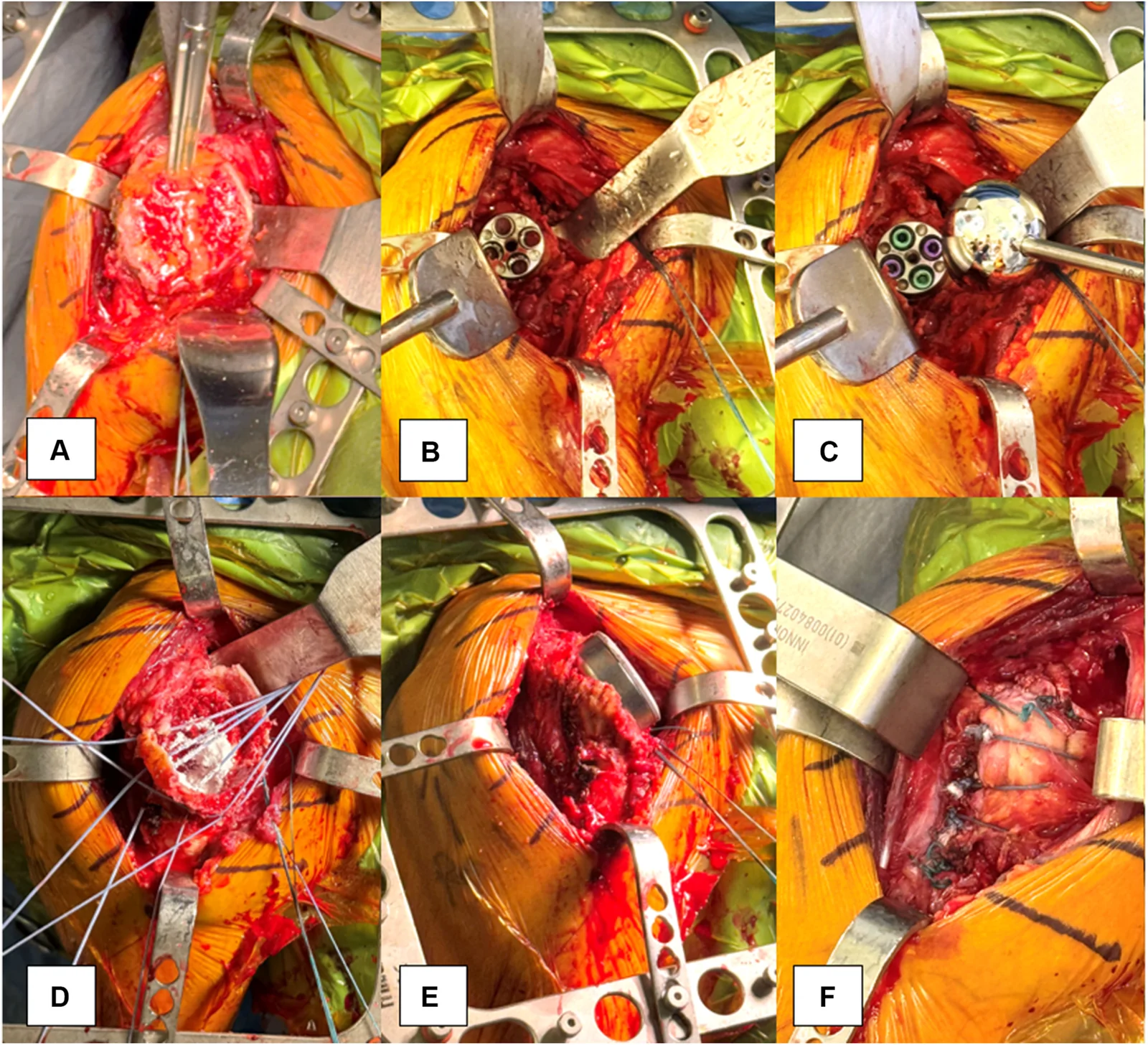
(**A**) Preparation of the humeral shaft, (**B**) Preparation of the glenoid, beginning with placement of a K-wire in the center of the glenoid, (**C**) Application of the glenosphere onto the baseplate, (**D**) After final shaft preparation, vancomycin powder is applied to the shaft, and transosseous sutures are placed for subsequent subscapularis repair, (**E**) Final humeral shaft construct, (**F**) Subscapularis repair performed using a modified Mason–Allen technique.

### Rehabilitation protocol

Post-operatively, patients wore an abduction sling for 3 weeks and were permitted early passive range of motion exercises. If a subscapularis repair was performed, external rotation was limited to 30° for the first 3 weeks to protect the repair. Active and active-assisted motion were initiated after 3 weeks.

### Patient-reported outcome measures and clinical evaluation

Demographic data, including sex, age at surgery, and hand dominance, were collected pre-operatively. Patient-reported outcome measures (PROMs) were collected pre-operatively and at final post-operative follow-up, with a minimum follow-up of 2 years. The American Shoulder and Elbow Surgeons (ASES) score, the Minimal Clinically Important Difference (MCID) for the ASES, and the patient acceptable symptom state (PASS) for the ASES were assessed. MCID was defined as a gain of a minimum of 11.4 points. The PASS was achieved if at least 80.8 points were achieved.^6^ Additional PROMs included the visual analog scale (VAS) for pain, the Single Assessment Numeric Evaluation (SANE), the Quick Disabilities of the Arm, Shoulder, and Hand (QuickDASH) questionnaire, as well as the Physical Component Summary and Mental Component Summary of the 12-Item Short Form Health Survey (SF-12).

Subjective satisfaction was rated on a 10-point Likert scale, where 10 indicated maximum satisfaction. Further clinical data included assessment of whether subscapularis repair was performed, assessment of fatty infiltration of the teres minor (as measured using the Goutallier classification system), and prior rotator cuff surgery. Pre-operative fatty infiltration of the teres minor muscle was assessed by 2 independent raters (B.C.M. and L.F.) on pre-operative MRIs.

### Multivariate analysis

An analysis of independent factors influencing post-operative outcomes—including pain, ASES, QuickDASH, and SANE scores—was performed. The factors analyzed comprised sex, age at surgery, subscapularis repair, pre-operative fatty infiltration, surgery on the dominant shoulder, and prior rotator cuff surgery. Patients were stratified according to the degree of fatty infiltration based on the Goutallier classification. Fatty infiltration was dichotomized into low-grade (grades 0–2) and high-grade (grades 3–4) categories. In addition, the impact of fatty degeneration on post-operative outcomes was analyzed for each Goutallier grade.

### Failures

Failures and complications were recorded. Failure was defined as any revision surgery involving the complete revision or exchange of the components of the arthroplasty. Complications were defined as any prosthesis-related adverse event managed either conservatively or with arthroscopic intervention. Also, any non-prosthesis-related adverse events, such as wound-healing issues, hematomas, and nerve injuries, were assessed.

### Statistical analysis

Statistical analysis was performed using IBM SPSS Statistics, Version 30.0 (IBM Corp., Armonk, NY, USA). Categorical variables were presented as counts and percentages. The Kolmogorov–Smirnov test was used to assess the normality of continuous data, which was additionally verified through visual inspection of histograms and Q–Q plots.

Normally distributed variables were reported as means with standard deviations, while non-normally distributed variables were presented as medians with interquartile ranges (IQR; 25th–75th percentile).

For comparisons between paired normally distributed variables, a paired Student's *t*-test was used, whereas the Wilcoxon signed-rank test was applied for paired nonparametric data. Differences between independent non-normally distributed groups were analyzed using the Mann–Whitney *U* test.

Multivariable linear regression analyses were performed to assess the influence of independent factors—including sex, age at surgery, subscapularis repair, pre-operative dichotomized fatty infiltration, surgery on the dominant shoulder, and prior rotator cuff surgery—on post-operative outcomes, such as pain, ASES, QuickDASH, and SANE scores. Linear regression was employed to evaluate relationships between each grade of fatty infiltration and post-operative ASES, QuickDASH, and SANE scores. A *P* value of <0.05 was considered statistically significant.

## Results

A total of 148 patients (80%) completed a final follow-up, with a mean follow-up duration of 76.1 ± 32.5 months. The mean age at surgery was 66.0 ± 9.8 years. Of these 148 patients, 91 patients (61.5%) were male. No significant sex difference was observed in age between male and female participants (65.2 ± 10.3 years vs. 66.1 ± 9.3 years; *P* > .05). Further demographic and surgical data are presented in Table I.

**Table I:** Patient demographics and surgical data.

Variable	Value
**Sex n (%)**	
Male	91 (61.5)
Female	57 (38.5)
**Age at surgery (yr, mean ± SD)**	66.0 ± 9.8
**Follow-up (mo, mean ± SD)**	76.1 ± 32.5
**Subscapularis repair n (%)**	121 (81.8)
**Previous rotator cuff surgery n (%)**	53 (35.8)
**Other indications than rotator cuff failure n (%)**	
Instability	5 (3.4)
Glenoid deformity	1 (0.7)
**Dominant shoulder affected n (%)**	89 (60.1)

*SD*, standard deviation.

Values are presented as mean ± standard deviation or number (percentage).

Patient demographics and surgical characteristics of the study cohort. Percentages are reported relative to the total cohort (N = 148). Continuous variables are presented as mean ± standard deviation, and categorical variables are presented as number (percentage).

Significant improvements were observed in ASES, VAS, SANE, QuickDASH, and SF-12 Physical Component Summary scores (*P* < .05). No significant improvement was detected in the SF-12 Mental Component Summary score. Additional details regarding PROMs are provided in Table II. Overall, 90.7% of patients reached the MCID for the ASES score, while 68.9% achieved the PASS.

**Table II:** Patient-reported outcome measures.

Parameter	Pre-operative	Post-operative	*P* value
ASES	48.3 (36.6-63.3)	88.3 (77.9-93.3)	<.001
Pain (VAS)	4 (3-6)	0 (0-1)	<.001
SANE score	49 (29-59)	89 (69-94)	<.001
QuickDASH	43.1 (34-56.8)	15.9 (9-34)	<.001
SF-12 PCS	38.9 (33.9-44.5)	50.6 (39.1-55.9)	<.001
SF-12 MCS	55.4 (45.9-60.9)	56.8 (49.5-59.0)	.6
Patient satisfaction	—	8 (5-10)	—

*ASES*, American Shoulder and Elbow Surgeons; *VAS*, visual analog scale; *SANE*, Single Assessment Numeric Evaluation; *QuickDASH*, Disabilities of the Arm, Shoulder and Hand; *SF-12*, 12-Item Short Form Survey; *PCS*, Physical Component Summary; *MCS*, Mental Component Summary.

Pre-operative and post-operative patient-reported outcome measures (PROMs) for the study cohort. Values are presented as medians (interquartile range) for non-normally distributed data. *P* values reflect paired comparisons between pre-operative and post-operative scores; a *P* value < .05 was considered statistically significant.

### Interrater reliability

Interrater reliability for the Goutallier classification was substantial, with an overall agreement of 80%. Disagreements occurred only between grades 1 and 2. The weighted Cohen's kappa was 0.80 (95% confidence interval [0.73, 0.85]), indicating substantial agreement between raters.

### Multivariate analysis

Multivariate analysis revealed no significant influence of most factors on ASES, VAS for Pain, SANE, or QuickDASH outcomes (*P* > .05). However, male sex was significantly associated with better outcomes in both SANE (male sex: 89 [69-94], female sex: 79 [49-94]) and QuickDASH (male sex: 13.6 [6.8-27.2], female sex: 22.7 [9.6-38.6]) scores (Tables III and IV). Furthermore, surgery on the dominant shoulder was significantly associated with improved QuickDASH scores (dominant shoulder: 13.6 [9-28.4] vs. non-dominant shoulder: 20.4 [10.2-38.6]) (Table III). No significant association was observed with fatty infiltration (*P* > .05).

**Table III:** Multivariable regression analysis predicting Q-DASH score.

Characteristic	β	95% CI	*P* value
SSC repair	−5.4	−12.5; 1.80	.141
Fatty infiltration	−1.7	−13.6; 10.2	.776
Prior RC surgery	−4.3	−10.9; 2.33	.203
Age at the time of surgery (yr)	−0.2	−0.6; 0.19	.319
Female sex	10.2	3.8; 16.7	**.002**
Dominant side affected	−9.2	−15.7; −2.7	**.006**

*β*, regression coefficient; *CI*, confidence interval; *SSC*, subscapularis; *RC*, rotator cuff; *Q-DASH* score = Quick Disabilities of the Arm, Shoulder and Hand Score.

Multivariable linear regression analysis identifying factors associated with post-operative QuickDASH scores. Regression coefficients (β) are presented with 95% confidence intervals (CIs); a *P* value < .05 was considered statistically significant. Significant *P* values are marked in bold letters.

**Table IV:** Multivariable linear regression analysis predicting SANE score.

Characteristic	β	95% CI	*P* value
SSC repair	7.1	−3.4; 17.5	.184
Fatty infiltration	−0.8	−18.2; 16.6	.929
Prior RC surgery	3.12	−6.6; 7.8	.526
Age at the time of surgery (yr)	0.1	−0.4; 0.7	.618
Female sex	−13.8	−23.4; −4.3	**.005**
Dominant side affected	7.2	−2.3; 16.7	.137

*β*, regression coefficient; CI, confidence interval; *SSC*, subscapularis; *RC*, rotator cuff; *SANE*, Single Assessment Numeric Evaluation. Multivariable linear regression analysis identifying factors associated with post-operative SANE scores. Regression coefficients (β) are presented with 95% confidence intervals (CIs); a *P* value < .05 was considered statistically significant. Significant *P* values are marked in bold letters.

### Failures and complications

A total of 7 failures (4.7%) occurred. The most common cause of failure was dislocation, occurring in 4 cases (2.7%), followed by infection in 2 cases (1.3%) and glenoid loosening in 1 case (0.7%). Six male patients (6.6%) and 1 female patient (1.8%) experienced a failure, which was not statistically significant (*P* = .25). The mean age for patients who experienced a failure was 70.3 ± 10.3 years, with only 1 patient younger than 65 years (the mean age of our cohort). In addition, 2 complications were observed (1.3%; 2 cases of acromial stress fracture, which were managed conservatively).

## Discussion

Our findings are consistent with our initial hypothesis that rTSA results in significant improvement in functional outcome scores and pain reduction at midterm follow-up. This is accompanied by low failure and complication rates and high patient satisfaction. Furthermore, surgery on the dominant arm and male sex were associated with superior outcomes in selected measures, whereas no other assessed factors demonstrated a significant influence on clinical results.

Consistent with current literature, our study demonstrated a significant improvement across several PROMs.^3^^,^^28^ Regarding the MCID and the PASS, our findings align with recent reports: 80%-100% of patients achieve the MCID, and 53%-73% reach the PASS for ASES.^26^^,^^27^ The comparatively higher MCID rates may be explained by lower pre-operative scores, which facilitated greater improvement but made attaining the absolute PASS threshold less likely.^26^

Female patients in our study were expected to achieve inferior outcomes on both the QuickDASH and SANE scores compared with male patients. Nevertheless, revision rates did not differ significantly between the 2 groups, which is consistent with prior research.^4^ As also noted in previous studies, female patients presented with lower pre-operative scores, which may partly account for the observed differences in post-operative outcomes.^18^^,^^30^

Reported complication rates in the literature include dislocations of up to 2.7%, acromial fractures of approximately 2.5%-5.3%, and infections of around 1.2%-1.6%.^7^^,^^15^^,^^20^^,^^21^^,^^23^ These values are consistent with our findings and underscore the overall low rate of post-operative complications and failures within our cohort and across the literature.^20^

This is the first study to demonstrate that QuickDASH scores are lower in the dominant extremity at midterm follow-up after rTSA. While the QuickDASH score is a well-established tool for monitoring post-operative recovery, previous studies have not identified superior functional outcomes or rehabilitation potential associated with surgery on the dominant vs. non-dominant side. Most of the existing literature, including studies on bilateral or side-to-side comparisons in shoulder arthroplasty, reports no meaningful differences in post-operative function, satisfaction, or pain between dominant and non-dominant shoulders. Of note, however, these studies did not report QuickDASH scores.^12^^,^^29^ Other factors that may affect functional outcomes include patient age at the time of surgery, comorbidities, and the severity of the pre-existing condition.^1^^,^^16^

Further research is warranted to explore the underlying mechanisms of recovery potential, as this may represent a key factor contributing to the superior rehabilitation outcomes observed for the dominant arm.

### Limitations

Several limitations should be acknowledged in this study. First, the retrospective review of prospectively collected data analysis carries an inherent risk of selection and reporting bias. Second, the evaluation of functional outcomes was not exhaustive, and range-of-motion measurements were not systematically recorded. Third, variability in follow-up duration across patients could have affected the outcome assessment. Fourth, linear multivariate regression assumes linear relationships, independence, homoscedasticity, and normally distributed residuals. Although these assumptions were reasonably met in our analysis, the method remains inherently limited, as it can only capture linear relationships and may therefore overlook more complex or nonlinear patterns in the data. Furthermore, the transition from a Grammont-style reverse shoulder arthroplasty design to a prosthetic design featuring +4 mm lateralization and a 135° neck-shaft angle may have influenced the observed outcomes.

Lastly, all surgeries were performed by a single surgeon, which may restrict the external validity of the findings but ensures consistency in surgical technique.

## Conclusion

rTSA provides consistent and durable clinical improvement, with high patient satisfaction and low complication and failure rates. Procedures performed on the dominant arm may yield more favorable outcomes, whereas female sex has been associated with inferior results in select domains, but without influencing overall failure rates.

## Disclaimers:

Funding: No funding was disclosed by the authors.

Conflicts of interest: Peter J. Millett reports royalties, consulting, and research support from Arthrex and equities in Vumedi. Both not related to this work. Any additional authors, their immediate families, and any research foundations with which they are affiliated have not received any financial payments or other benefits from any commercial entity related to the subject of this article.
